# Effect of Nutritional Antioxidants on Periodontal Disease and Periodontal Therapy

**DOI:** 10.3390/dj13120570

**Published:** 2025-12-02

**Authors:** Konstantina Vavetsi, Tzortzis Nomikos, Spyridon Vassilopoulos, Yiorgos A. Bobetsis

**Affiliations:** 1 Private Practice, 11527 Athens, Greece; kvavetsi@yahoo.gr; 2 Department of Nutrition and Dietetics, School of Health Sciences & Education, Harokopio University, 17676 Athens, Greece; tnomikos@hua.gr; 3 Department of Periodontology, School of Dentistry, National and Kapodistrian University of Athens, 11527 Athens, Greece; spvas@dent.uoa.gr

**Keywords:** antioxidants, copper, nutrition, periodontal disease, periodontal treatment, selenium, vitamin A, vitamin C, vitamin E, zinc

## Abstract

Oxidative stress plays a significant role in the pathophysiology of periodontal disease (PD). Therefore, it is reasonable to suggest that a diet rich in antioxidants, such as several vitamins and essential minerals, may positively affect periodontal health. However, the evidence from the relevant literature is yet inconclusive. Studies evaluating the levels of intake of nutritional antioxidants in relation to periodontal status demonstrate contradictory results. This inconsistency could be attributed to heterogeneity in study design and methodological limitations, such as the small sample size, the assessment of periodontal status based on partial mouth measurements that usually underestimate the actual severity of PD, the lack of adjustment for confounders, which may obscure any potential but weak effect of nutrition, and the use of a 24 h recall to assess nutrient intake. Regarding the intervention studies that evaluated the effect of nutritional antioxidant supplementation as an adjunct to periodontal therapy, again, the results are not consistent. Few studies are randomized, and often different nutritional supplements are combined, making it difficult to distinguish the actual effect of each nutrient. Moreover, the dosage and duration of use of these supplements vary, rendering comparisons impossible. Therefore, better designed studies are necessary for the future. The assessment methods used for both PD and vitamin/mineral intake need to be more accurate and standardized to improve comparability. Also, prospective longitudinal and randomized controlled studies are important to establish the optimal dosing and the long-term effects of vitamin/mineral supplementation on periodontal health in different patient populations.

## 1. Introduction

Periodontitis is the most common inflammatory disease, mainly caused by oral microorganisms, and is characterized by the progressive destruction of the supportive tissues of the teeth [1]. To control periodontal infection, the host mounts an immune response against the periodontopathogens, which triggers, among others, the release of reactive oxygen species (ROS), especially from neutrophils. It has been demonstrated that ROS play a substantial role in regulating the differentiation of osteoclasts [2]. In addition, current evidence suggests their role in the pathogenesis of fatty degenerative osteonecrosis of the jaw [3]. Regarding periodontal disease, the increase in free radicals induces a state of oxidative stress, which is associated with more severe periodontal destruction [4,5]. The meta-analysis by Mohideen et al. [5] demonstrated that patients with periodontitis exhibit significantly higher levels of total oxidative stress and lower total antioxidant capacity compared to healthy controls, revealing that an imbalance between oxidants and antioxidants plays a critical role in the onset and progression of periodontitis. Malondialdehyde (MDA), 8-hydroxy-deoxyguanosine (8-OH-dG), and nitric oxide (NO) are all substances related to oxidative stress. Τhe meta-analysis of Chen et al., 2019 [6], concluded that there is a significant decrease in TAC and a significant increase in MDA, NO, total oxidant status, and (8-OH-dG) in the saliva of patients with periodontitis. Periodontitis patients also demonstrated an elevation of MDA levels in the gingival crevicular fluid. Besides periodontal disease (PD), smoking and diabetes mellitus are also capable, among others, of causing oxidative stress [7].

Protection against oxidative stress relies mainly on endogenous antioxidant enzymatic systems, endogenous small organic molecules, and dietary micronutrients that scavenge and neutralize ROS [8]. Dietary antioxidants (vitamins, minerals, trace elements) can scavenge free radicals, support endogenous antioxidant systems by serving as co-factors of antioxidant enzymes, regenerate oxidized vitamins, and act as precursor molecules for the cellular production of endogenous antioxidants [9]. In humans, the role of nutrition is critical for this process, as most of the antioxidants are obtained through the diet. Healthy nutrition, mainly in terms of adherence to plant-based diets, includes several components rich in antioxidant properties. These micronutrients are required only in small quantities and consist mainly of the antioxidant vitamins A, C, and E, as well as the minerals copper (Cu), zinc (Zn), and selenium (Se), which serve as coenzymes of the main antioxidant enzymes, such as glutathione peroxidases and superoxide dismutases [10].

To date, many investigators have tried to evaluate whether the level of intake of these antioxidants may affect periodontal tissue breakdown and whether supplementation with antioxidants as an adjunct to non-surgical periodontal therapy (PT) would offer an additional benefit. Against that background, the aim of this review is to critically present the current evidence regarding the effect of nutritional antioxidants on PD and PT.

## 2. Methodology

The literature search was conducted using the databases PubMed and Scopus, with the search being limited to studies published up until March 2025. The keywords and MeSH terms used were “Vitamin A”, “retinol”, “carotenoids”, “Vitamin C”, “ascorbic acid” “Vitamin E”, “tocopherol”, “copper”, “zinc”, “selenium”, “antioxidants”, “periodontal health”, “periodontitis”, “gingivitis”, “periodontal disease”, and “oxidative stress”.

The inclusion criteria encompassed studies involving human adult participants (age ≥ 18 years). The studies included were observational (cohort, case–control, cross-sectional) or supplementation trials, and other relevant clinical studies. The dietary intake should be assessed either by 24 h recall, dietary records, or food frequency questionnaires (FFQs), and the status of each vitamin or trace element by specific biochemical measurements. Periodontitis was assessed by several periodontal parameters, such as probing pocket depth (PPD) changes, clinical attachment loss (CAL), and indices representative of gingival inflammation, like bleeding on probing (BoP).

Articles that were not published in English, animal studies, in vitro and ex vivo studies, studies focusing on multivitamin supplements that could not evaluate the effects of each vitamin or mineral separately, and studies that did not report periodontal outcomes as reported above were excluded from the present review.

Data were manually extracted from the selected studies. As primary data points of interest, we considered the key clinical indicators of periodontal health, like the gingival index, pocket depth, and clinical attachment levels.

In Table 1, we present the dietary reference values according to the European Food Safety Authority [11], so that the readers of this review can better assess the clinical significance of the outcomes presented in the next chapters.

## 3. Vitamins

### 3.1. Vitamin A

Vitamin A is a fat-soluble vitamin that encompasses a group of chemically related organic compounds with multiple functions, such as the support of cell growth and differentiation, regulation of immune response, and maintenance of healthy vision [12]. The human diet contains two sources of vitamin A: preformed vitamin A (retinol and retinyl esters) and provitamin A carotenoids (alpha-carotene, beta-carotene, and beta-cryptoxanthin). Preformed vitamin A is found in foods from animal sources, including dairy products, eggs, fish, and organ meats, while provitamin A carotenoids are plant pigments [13,14,15].

The antioxidant activity of vitamin A and carotenoids is related to the hydrophobic chain of polyene units that can quench singlet oxygen, neutralize thiyl radicals, and combine with and stabilize peroxyl radicals. Vitamin A and carotenoids can autoxidize when oxygen tension increases and thus are more effective antioxidants at low oxygen tensions typically found in physiological tissues [13].

Regarding cross-sectional studies (Table 2) and beginning with the older ones, an international nutritional study in 1963 [16] including more than 21,000 participants demonstrated that populations with high scores for PD tended to be deficient in vitamin A. On the other hand, the study of Waerhaug et al. [17], which recruited a large sample of 8217 subjects 13–60 years old in Ceylon, found that vitamin A deficiency, assessed by severe clinical symptoms (xerosis, keratitis, xeropthalmia, blindness, phrynoderma), was not associated with elevated periodontal indices.

Freeland et al., in 1976 [27], recruited 80 dental patients and found a slight inverse relation of vitamin A with the periodontal index (PI). However, the results were based on just one 24 h dietary recall, which by design has several limitations [28].

In another large cross-sectional study by Park et al., 2017 [18], involving 2049 young Korean adults aged 19–39 years, vitamin A intake was evaluated from 24 h recall. After adjustment for several confounders, multivariate logistic regression analysis revealed that female participants with a vitamin A intake lower than the median value had a marginally higher, but non-significant risk of periodontitis compared with women consuming vitamin A above the median value (Odds Ratio (OR): 1.56; 95% Confidence Interval (CI): 1.00–2.44).

The cross-sectional study of Hosoda et al., 2021 [19], which included 120 female college students, reported a lower intake of beta-carotene in periodontitis patients compared to heathy participants (1515 μg vs. 2068 β-carotene equivalents/1000 Kcal, *p* < 0.05). However, the multivariate logistic regression analysis, using the Body Mass Index (BMI), presence of snacks, and dietary hardness as confounders, showed no significant association between vitamin A intake and PD.

Watson et al., in a cross-sectional study [29], examined the impact of single-nutrient intake based on 24 h dietary recall on up to five separate occasions over 16 months. A higher intake of the “high micronutrient and fiber” dietary pattern was associated with a lower risk of PD. However, in the single-nutrient analysis, β-carotene was not associated with a decreased risk of PD after adjustment for sociodemographic and health behavior variables. It should be noted, however, that in this study, PD was self-reported, and the participants used a touchscreen questionnaire indicating the presence of gingival bleeding or pain or loose teeth. Thus, although the study sample size was large (9476 participants), the subjectivity in periodontal data collection is a major concern, as no clinical examination was performed.

There are several studies investigating the relation of nutrition and periodontitis using data from the NHANES cohort. Luo et al., 2018 [20], used data from 2011 to 2014. Daily micronutrient intake was obtained from files of 24 h individual food dietary recall interviews. The authors observed that the adjusted OR for having periodontitis was 1.78 for those consuming insufficient amounts of vitamin A (≤61 μg/day) compared to those consuming ≥ 786 μg/day. Irrespective of the large sample size (6415 participants), the recall interview of just one day remains an important limitation.

Li et al. (2022) [21] evaluated associations between micronutrient intake and periodontitis using data from 8959 participants of NHANES (2009–2014). A marginally significant inverse association of vitamin A intake and risk of periodontitis was observed, while a dose–response effect examination revealed an almost linear, inverse association of vitamin A intake with the risk of periodontitis. The authors calculated that the sufficient intake of vitamin A, able to reduce the risk of periodontitis, was 527 μg retinol activity equivalents/day. The same group [30], using data from the NHANES study from 2009 to 2014 (8959 participants) with two complete records of 24 h dietary intake, found that a high α-carotene intake ≥ 55.8 μg/day was associated with a lower risk for periodontitis (adjusted OR: 0.70, 95% CI: 0.53–0.91, *p* = 0.010) compared to a low α-carotene intake ≤ 55.8 μg/day in patients with type II diabetes. Finaly, Zhou et al., 2023 [22], in a cross-sectional study from NHANES with 9081 participants, concluded that compared with the lowest tertile, individuals in the highest tertile of retinol intake were less likely to have periodontitis (OR: 0.79, 95% CI: 0.65–0.96). This association remained significant even in populations that were younger than 60 years old, non-Hispanic Black, non-smokers, obese, non-diabetic, and non-hypertensive.

On the other hand, results derived from the study of Liang and co-workers [23], which included 9820 subjects aged ≥ 30 years from the 2009–2014 NHANES, did not find an association between vitamin A intake and periodontitis risk after adjusting for several factors. In addition, the study by Chen and co-workers [24], who used the 2009–2014 NHANES data including two 24 h recalls, also observed that vitamin A was not associated with periodontitis.

Several groups have also studied the association between vitamin A status and periodontitis. The assessment of vitamin A status was mainly determined from the serum levels of vitamin A. The study by Chapple and co-workers [25] examined the relationship between serum vitamin A status and periodontal health in 11,480 adult subjects from the NHANES III. After adjustment for several factors, an inverse association between serum vitamin A levels and the prevalence of periodontitis could not be confirmed. Serum levels of micronutrients were also evaluated in the study by Linden et al., 2009 [26]. The authors examined 1258 men from Northern Ireland aged 60–70 years for α- and β-carotene and β-cryptoxanthin. Models were constructed using two measures of periodontal status (low- and high-threshold periodontitis) as dependent variables and the quintiles of each antioxidant as a predictor variable. The authors observed that low levels of carotenoids were associated with a significantly increased risk of moderate (low-threshold) periodontitis. Only β-carotene and β-cryptoxanthin were associated with an increased risk of generalized severe (high-threshold) periodontitis after adjusting for major confounders. Regarding retinol, no significant associations were found.

In an effort to quantify the overall effect of vitamin A intake on PD status, two recent meta-analyses have been conducted. In the first, by Mi et al., 2024 [31], four observational studies including a total of 17,642 healthy individuals and 7832 patients with PD were included. The authors found that higher levels of vitamin A intake were negatively correlated with PD (OR: 0.788, 95% CI: 0.640–0.971). Interestingly, although a significant heterogeneity between studies was observed (I^2^: 79.1%, *p* = 0.003), leave-one-out sensitivity analysis demonstrated that the overall results did not change substantially after excluding any one study. The meta-analysis of Luca et al., 2024 [32], which included six cross-sectional studies (50,722 participants), demonstrated a more modest association of vitamin A intake and periodontitis risk (pooled OR: 0.97, 95% CI: 0.94–1.00).

Gao et al., 2024 [33], conducted a two-sample Mendelian Randomization (MR) analysis regarding the relationship of diet-derived circulating antioxidants with periodontitis and dental caries and performed Inverse Variance Weighted (IVW) analysis separately in different databases, followed by meta-analysis. According to the IVW analysis, among all other circulating antioxidants, only the elevated levels of circulating retinol reduced the risk of periodontitis [pooled OR for periodontitis risk per unit increase in absolute circulating retinol was 0.300 (95% CI :0.15–0.61, *p* = 0.001; Heterogeneity: I^2^: 40.3%, *p* = 0.196)].

Finally, the role of β-carotene in periodontal healing was assessed by the clinical trial of Dodington et al., 2015 [34], in 23 smokers and 63 non-smokers. Eight to sixteen weeks after non-surgical PT, probing pocket depth (PPD) reduction was significantly associated with β-carotene intake, as evaluated by block FFQs, only in the non-smoking group. The authors found that associations between β-carotene and periodontal healing were not detected in smokers either due to the small sample size or because smoking is an overwhelming risk factor for PD, which compromises oral wound healing. In addition, studies in humans show that smokers have lower circulating levels of β-carotene [35], making it possible that associations were not observed due to a depletion of antioxidants in smokers as a result of oxidative turnover. It should also be noted that although this study demonstrates the beneficial effect of a higher β-carotene intake in periodontal healing, to date, there are no intervention studies that assess the effect of vitamin A supplementation as an adjunct to non-surgical PT. Studies using a combination of micronutrients as supplements cannot distinguish the effect of each individual component and therefore were not included in this review.

The aforementioned studies have clearly shown that a sufficient intake of vitamin A can minimize the risk of periodontitis. Although there is a scarcity of studies investigating the mechanisms by which vitamin A can interact with the pathophysiology of periodontitis, it seems that its ability to maintain the epithelial integrity, its antioxidant properties, and its immunomodulatory roles can partly explain its periodontitis protective properties [36,37]. Very recently, a genetic bioinformatics study demonstrated a novel role of vitamin A, which is the restoration of metabolic reprogramming of macrophages via the JAK-STAT pathway. Metabolic reprogramming of macrophages (transition from oxidative phosphorylation to glycolysis, Warburg effect) is a process that augments inflammation and impairs the regenerative capacity of the periodontal tissues [38].

As summarized, both epidemiological and preclinical studies indicate the protective effect of vitamin A against periodontitis. However, high-quality randomized clinical trials are needed that focus both on the prevention and treatment of periodontitis to determine the extent to which vitamin A may play a significant role in managing the condition.

### 3.2. Vitamin C

Ascorbic acid (AscH2), better known as vitamin C, is a water-soluble ketolactone with two ionizable hydroxyl groups. The dominant form at the physiological pH is the ascorbate monoanion (AscH−), which is an excellent reducing agent and readily undergoes two consecutive one-electron oxidations to form an ascorbate radical and dehydroascorbic acid (DHA). Vitamin C also promotes collagen biosynthesis by preventing iron-dependent oxidation and auto-inactivation of lysyl and prolyl hydroxylase [39]. Plants and most animals synthesize ascorbate from glucose. However, humans lack this capability and hence receive ascorbate and DHA through their diet [40,41,42]. The role of ascorbic acid deficiency in PD has been acknowledged since the 18th century, when scurvy was associated with gum bleeding in sailors. In accordance with these initial observations, Jacob et al., 1987 [43], found that vitamin C deficiency results in gingival bleeding, regardless of the level of oral hygiene. Indeed, gingival bleeding reduction was observed after normal (65 mg/day) ascorbic acid intakes as compared to deficient (5 mg/day) intakes and after supplementary (605 mg/day) intakes as compared to normal intakes. The crucial role of vitamin C in reducing gingival bleeding has been attributed mainly to its anti-histamine properties, which prevent the increase in capillary permeability and therefore of bleeding and tissue edema.

In more recent studies, a lower concentration of vitamin C was observed in patients with gingivitis [44,45], while vitamin C deficiency was associated with the presence of necrotizing ulcerative gingivitis [46]. In 1992, Blignaut and Grobler [47] employed the Community Periodontal Index of Treatment Needs (CPITN) to compare the periodontal status of fruit-farm workers, who consumed large amounts of fresh fruits, with workers on grain farms. Workers consuming citrus showed a significantly lower prevalence of deep periodontal pockets (CPITN codes 3 and 4) although they exhibited significantly less healthy periodontal sextants compared to the other groups. It is noteworthy that the CPITN index partially evaluates periodontal status and hence is selected in larger epidemiological studies for practical reasons. However, the results from these studies need to be interpreted with caution, since the CPITN index tends to underestimate PD severity [48].

The cross-sectional study by Lee and co-workers [49] evaluated data from 10,930 Koreans of the Korean NHANES IV. The authors demonstrated that higher intakes of vitamin C were associated with a lower risk of PD. Specifically, based on a 24 h dietary record and after adjusting for confounders, participants with an inadequate vitamin C intake (<75 mg/day) were 1.16 times more likely to have periodontitis than those with an adequate dietary vitamin C intake. On the other hand, Hosoda and co-workers 2021 [19] found no statistically significant differences concerning vitamin C intake between the PD group and the non-PD group.

The study by Park et al. (2017) found that young adults consuming less than the RNI (100 mg/day) of vitamin C had a higher risk of periodontitis compared to those whose intake exceeded the RNI, particularly among women and non-smokers [18]. In addition, the aforementioned study of Chapple et al. from the NHANES III showed that participants in the highest quintile of serum vitamin C (70.4 mmol/L) had an OR of 0.61 (95% CI: 0.49–0.74) and 0.53 (95% CI: 0.42–0.68) to have mild and severe periodontitis compared to the participants of the first quintile (8.52 mmol/L). The effect was more pronounced [0.38 (0.26,0.63)] for never-smokers [25].

Cross-sectional studies were also conducted based on the US NHANES data. In particular, Ismail et al., 1983 [50], using data from the NHANES I, demonstrated a significant, but weak inverse correlation between dietary vitamin C intake and PPD after controlling for possible confounders, including oral hygiene. In this study, vitamin C dietary intake was based on a 24 h recall interview. Nishida et al., 2000 [51], and Luo et al., 2018 [20], based on NHANES data, concluded that there was a dose-dependent inverse relationship between the levels of dietary vitamin C and PD severity. On the contrary, Li et al., 2022 [21], using data from the 2009–2014 NHANES, which assessed nutrient intake by internal interview, found a linear positive association between vitamin C intake and periodontitis. Similarly, Chen and co-workers [24], and Liang and co-workers [23] using the same NHANES dataset, found that vitamin C was not causally associated with periodontitis.

Several studies have also evaluated the effect of the vitamin C status by measuring its concentration in the blood. Vitamin C is fully acquired by food consumption, and therefore this method is more reliable since possible errors of omission or intrusion, which are common in questionnaires, are avoided. In this set of studies, all found that higher levels of vitamin C are associated with a lower risk of periodontitis [52,53]. A similar pattern was also observed when individual PD parameters were assessed. Specifically, Amarasena et al., 2005 [54], examined serum vitamin C levels of 413 70-year-old Niigata citizens and observed an inverse relationship with clinical attachment loss (CAL) at a bivariate level (r: −0.23, *p* < 0.00005). Multiple regression analysis demonstrated that the association of serum vitamin C levels and CAL remained significant in a model that included smoking, diabetes mellitus, oral hygiene, gender, or number of teeth present as confounders. The association was weak (correlation coefficient ± SE: −0.04 ± 0.02, *p* < 0.05) but statistically significant in this elderly population. Table 3 summarizes the aforementioned cross-sectional studies.

A small Indonesian cohort study (N = 128) found an inverse correlation between the plasma vitamin C level and periodontal attachment loss [55].

The cohort study of Iwasaki et al., which assessed the vitamin C intake using the FFQ [56] and serum concentration [57], found a longitudinal relationship between vitamin C and PD. In particular, with regard to the FFQ, the multivariate-adjusted incidence rate ratios for PD were 0.76 (95% CI: 0.60–0.97) and 0.72 (95% CI: 0.56–0.93) for the second (80.5–100.4 mg/day) and third tertiles (100.4–195.6 mg/day) of vitamin C concentration, respectively. Similarly, concerning the serum concentration of ascorbic acid, the multivariate adjusted relative risks for PD were 1.12 (95% CI: 1.01–1.26) and 1.30 (95% CI: 1.16–1.47) in the middle (6.0–8.5 μg/mL) and lowest tertiles (0.2–5.9 μg/mL), respectively, compared to highest (8.6–22.6 μg/mL). Both analyses support the hypothesis that a low dietary intake and serum levels of vitamin C may be a risk factor for PD in the Japanese elderly.

Regarding case–control studies, that of Väänänen et al., 1993 [58], compared 75 cases with low vitamin C plasma levels with 75 controls (≥50 mumol/L) matched for age, gender, and number of teeth. The authors concluded that the proportion of sites with bleeding on probing and PPD ≥ 4 mm was significantly higher, almost double, in the case group than in the controls.

Concerning clinical trials, in the study of Staudte et al., 2005 [59], 58 patients with chronic periodontitis were assigned to a test group (21 non-smokers; 17 smokers) and a diseased control group (11 non-smokers; 9 smokers), and 22 healthy individuals were also recruited in the study. At baseline, significantly reduced plasma vitamin C levels were observed in the test group and diseased controls compared to healthy controls. Following grapefruit consumption, the mean plasma vitamin C levels rose significantly in the test group in comparison with the diseased controls (non-smokers: 0.87 ± 0.39 mg dL, smokers: 0.74 ± 0.30 mg dL). In addition, the Sulcus Bleeding Index (SBI) was reduced in the test group (non-smokers: from 1.68 ± 0.6 to 1.05 ± 0.6, *p* < 0.001). On the other hand, no changes were observed regarding Plaque Index and PPD. The authors concluded that periodontal patients, especially smokers, exhibit plasma vitamin C levels below the normal range [59].

Gokhale and co-workers concluded that the plasma ascorbic acid level was below the normal range (0.77 ± 0.38 mg/dL) in systemically healthy subjects with gingivitis (0.40 ± 0.39 mg/dL) and diabetics with periodontitis (0.37 ± 0.25 mg/dL) and that dietary ascorbic acid supplementation with scaling and root planing (SRP) improved the SBI in subjects with gingivitis and diabetics with periodontitis [60].

Abou Sulaiman et al. recruited 30 patients with periodontitis (15 patients received non-surgical treatment with an adjunctive dose of vitamin C and 15 patients received non-surgical periodontal treatment alone) and 30 matched controls. Following non-surgical periodontal treatment, periodontal parameters improved among both case groups (*p* < 0.001). Nevertheless, the adjunctive dose of vitamin C did not provide an additional effect (*p* > 0.05) [61].

Woolfe et al., 1984, in another intervention study, observed no significant differences between the vitamin C and the placebo group, suggesting that the use of high doses of vitamin C (250 mg/day) in nondeficient individuals does not have a predictable or strong effect on the gingival response to periodontal therapy [62].

Similarly, Vogel et al., 1986, who also examined the effects of megadoses of vitamin C (1500 mg/day) on gingival health, found no significant differences concerning responses to experimental gingivitis and polymorphonuclear neutrophil chemotaxis [63].

Some interesting findings have also been reported in smokers. Smoking is known to deplete vitamin C levels because of the enhanced oxidative stress it causes in the body. Since vitamin C is a potent antioxidant, it can scavenge oxidants generated in cigarette smoke, but the vitamin becomes consumed in this process [64]. In accordance with these observations, serum vitamin C levels were lower in smokers with periodontitis compared with both periodontally healthy non-smokers and non-smokers with periodontitis [65].

Besides the beneficial effect of vitamin C on clinical periodontal parameters, Pussinen et al., 2003 [66], found a negative correlation with serum antibodies against the periodontopathogens *A. actinomycetemcomitans* and *P. gingivalis*. In addition, according to Staudte et al., 2010 [67], vitamin C reduced the cytotoxic and apoptotic effects of *P. gingivalis* on human gingival fibroblasts in vitro. Finally, it is noteworthy that a single-nucleotide polymorphism, in particular rs6596473 in the gene SLC23A1, which codes for a transmembrane vitamin C transporter, was associated with aggressive periodontitis in a German and a Dutch population [68,69].

The role of vitamin C supplementation as an adjunct to non-surgical periodontal therapy has also been examined. Specifically, the effect of 500 mg/day vitamin C supplementation on periodontitis patients with uncontrolled type 2 diabetes mellitus after non-surgical PT was evaluated in a double-blind, placebo-controlled, clinical trial [70]. Although all periodontal parameters were significantly improved from baseline in both groups, no significant difference was found between groups. The study had a good design and a well-documented sample size calculation. Nevertheless, the results cannot be generalized, since diabetic patients demonstrate higher turnover rates for vitamin C and abnormalities in its metabolism [71].

### 3.3. Vitamin E

Vitamin E is a lipophilic vitamin comprising a group of eight compounds, four tocopherols (αT, βT, γT, and δT) and four tocotrienols (αTE, βTE, γTE, and δTE), which are related in molecular structure [72]. Vitamin E cannot be synthesized in the human body and therefore must be supplied exogenously through the diet [73]. Vitamin E exhibits various potentially beneficial effects on human health, such as anti-lipidemic [74], anti-obesity [75], and anti-inflammatory [76] effects. In addition, vitamin E is a potent antioxidant with lipoperoxyl radical scavenging activities. αT is the most abundant form of vitamin E in tissues, and a low intake of this form results in vitamin E deficiency-associated ataxia [77,78]. Human symptoms of vitamin E deficiency suggest that its antioxidant properties play a major role in protecting erythrocyte membranes and nervous tissues [79]. αΤ is the only form of vitamin E that is maintained in human plasma and is used for the estimation of vitamin E requirements [80].

The effect of vitamin E on PD has been examined for decades. In 1976, Slade et al. [81] found no significant difference in serum vitamin E levels between 12 periodontal patients and 12 controls. However, the extremely small sample size significantly attenuates the statistical power of this study. On the contrary, Hosoda and co-workers, 2021 [19], in their cross-sectional study, observed, in both univariate and multivariate analyses, that vitamin E intake in the PD group (4.1 ± 1.0 mg/1000 Kcal) was significantly lower than in the non-PD group (4.7 ± 1.1 mg/1000 Kcal). In the same direction, the cross-sectional study of Watson and co-workers, 2022 [29], also found that higher intakes of vitamin E were associated with a lower risk of PD.

Several studies have also evaluated data deriving from the US NHANES. Specifically, Chapple et al., 2007 [25], used data from the NHANES III and found no significant difference between vitamin E levels and periodontitis. Similar results were also observed by Chen and co-workers [24], who assessed data from the 2009–2014 NHANES. In contrast, Zong et al., in 2015 [82], examined 4708 participants from the 1999–2001 NHANES, where serum tocopherols were measured by high-performance liquid chromatography and values were adjusted for total cholesterol (TC) and other confounders. Non-linear inverse associations of serum αT with CAL, PPD, and total periodontitis (TPD) were observed, which were restricted to adults with a normal but relatively low αT status. In addition, participants with a γT:TC ratio in the interquartile range showed a significantly lower mean PPD than those in the highest quartile. Using data from NHANES, Luo et al., 2018 [20], also demonstrated that a low vitamin E intake (≤4.54 mg/day vs. ≥11.11 mg/day) was associated with an increased severity of PD (adjusted OR: 1.576). Likewise, the cross-sectional NHANES study of Li and co-workers [21] concluded that the vitamin E intake was inversely correlated with the periodontitis risk in a complex, non-linear manner. In fact, multifactorial logistic regression showed that an intake within the recommended range of vitamin E levels reduced the risk of periodontitis. Finally, the cross-sectional study of Liang et al., 2024 [23], using again data from the 2009–2014 NHANES reported that higher intake levels of vitamin E (≥7.48 vs. <7.48 mg/day) were related to a decreased likelihood of developing periodontitis compared to lower intake levels (OR 0.79, 95%CI: 0.69–0.92, *p* = 0.004). Table 4 summarizes the aforementioned cross-sectional studies.

The cohort studies by Iwasaki in 2013 [56] and in 2012 [57] also reported an inverse association of high vitamin E intake with the number of teeth with PD progression and a significant association of a low serum level of αT with a higher number of PD events. Hence, the authors concluded that low serum levels of αT may be a risk factor for PD in the Japanese elderly.

Finally, the recent systematic review and meta-analysis by Bumbu et al., 2024 [83], revealed a heterogeneous response to tocopherol supplementation, with a pooled odds ratio for efficacy in reducing PD severity of 0.97 (95% CI: 0.96–0.98). When vitamin E was insufficient, a statistically significant increase in CAL and PPD was observed, with odds ratios ranging from 1.15 to 9.33. Nevertheless, variations in tocopherol’s effectiveness were highlighted by the considerable heterogeneity (I^2^ = 88.35%).

Regarding studies exploring the role of αT in periodontal healing following non-surgical periodontal therapy, the previously discussed study by Dodington and co-workers [34] found that in non-smokers, a higher dietary intake of αΤ through the diet and supplements was significantly associated with reduced PPD after scaling and root planing (SRP).

Regarding clinical trials, the effect of vitamin E supplementation as an adjunct to PT has been evaluated in two intervention studies. The first study, by Singh et al. in 2014 [84], randomly assigned 38 patients to one of two treatment groups. The first group received SRP and the second SRP along with vitamin E supplementation (200 mg every two days) for 3 months. At the 3-month follow-up a higher improvement in periodontal parameters was observed in the second group. The researchers suggested that adjunctive vitamin E supplementation improves periodontal healing, as well as superoxide dismutase (SOD) activity, indicative of antioxidant defense. In the second clinical trial, by Behfarnia et al., 2021 [85], sixteen periodontitis patients were divided into two groups. The case group received SRP and vitamin E supplementation (200 IU daily for up to 2 months), while the control group received SRP alone. Although intergroup PPD changes were not significant after 2 months, CAL significantly decreased only in the case group, indicating the additional benefits of vitamin E supplementation on non-surgical PT.

## 4. Minerals

### 4.1. Copper

Copper (Cu), manganese (Mn), and zinc (Zn) are part of the group of superoxide dismutase enzymes (MnSOD, Cu/ZnSOD), which catalyze the superoxide anion dismutation into hydrogen peroxide and oxygen. The hydrogen peroxide formed is then decomposed into water and oxygen by catalase or glutathione peroxidase. Therefore, superoxide dismutase is an antioxidant enzyme that neutralizes harmful oxygen free radicals. The role of the above minerals in this process is crucial, since even a small change in their level in the tissues causes a disturbance in enzyme metabolism [86].

Needed only in trace amounts, the human body contains approximately 100 mg of Cu. As a transition metal, it is a cofactor of many redox enzymes, ceruloplasmin being the most abundant Cu-dependent ferroxidase enzyme with a Cu-dependent oxidation activity. Apart from its role in iron metabolism, the need for Cu also derives from its involvement in many biological processes, including antioxidant defense, neuropeptide synthesis, and immune function [87,88].

Elevation of serum Cu is observed during a variety of stress conditions, including inflammation [89]. Freeland et al., 1976 [27], in their cross-sectional study, examined 80 non-fasting individuals and recorded a periodontal index (PI), based on visual and radiographic data, and also included a 24 h dietary recall. The serum Cu level was linearly related (r = 0.64; *p* < 0.001) to the PI, but the dietary intake of copper was not related to PI or serum Cu levels. Therefore, the authors concluded that the increase in serum Cu concentration was not influenced by dietary intake, but possibly by inflammation. However, these results need to be interpreted with caution, due to several methodological limitations, such as the small sample size, the PI used, and the single 24 h recall to assess Cu intake from food.

In the more recent literature, the cross-sectional study by Hamasaki and co-workers, 2017 [90], recorded the Community Periodontal Index (CPI) as an index of PD for 3043 participants using linked data from the 2005 National Health and Nutrition Survey, the Comprehensive Survey of Living Conditions, and the Survey of Dental Diseases in Japan. The diet survey period was again only one day; however, the researchers tried to avoid periods in which the diet may have changed. According to univariate analyses, subjects in the group with CPI 3–4 had significantly higher Cu levels than those with CPI 0–2. Nevertheless, multivariate analysis showed no association.

The case–control study of Yu et al., 2025 [91], examined 14 micronutrients, including Cu, in the serum of 4784 PD cases and 272,252 controls. Pooled data were collected from genome-wide association study (GWAS) and publicly available data. These pooled data were analyzed by two-sample Mendelian randomization to determine whether a causal relationship exists between serum micronutrients and periodontitis. No significant association between Cu and periodontitis was found.

Finally, although there are no studies investigating the effect of Cu supplementation alone as an adjunct to PT, there is one intervention study [92] that examined the effect of non-surgical PT on the serum Cu concentration and glycemic status. A total of 120 subjects were equally divided into three groups: group 1—patients with periodontitis, group 2—patients with periodontitis and controlled diabetes, and group 3—patients with periodontitis and uncontrolled type 2 diabetes. Smokers were excluded from this study. Periodontal parameters were evaluated at baseline and 3 months after PT. Intergroup comparisons demonstrated that, at baseline, group 1 had the lower Cu levels. In addition, as expected, periodontal intervention improved periodontal indices and glycemic control. Moreover, Cu serum levels decreased in all groups, but changes were significant only in group 1. The authors linked the reduction in Cu levels after PT to the dampening of inflammation and suggested that high levels of Cu could alter collagen metabolism and promote tissue breakdown in periodontitis.

### 4.2. Zinc

As already described, Zn, along with Cu and Mn, is part of the group of superoxide dismutase enzymes (MnSOD, Cu/ZnSOD). Its concentration has a significant influence on the activity of antioxidant enzymes and thus is critical against oxidative stress [86]. Zn belongs to the divalent metals in group 12 of the periodic table and, unlike other bioactive metals such as iron (ferric state (Fe^3+^) or ferrous state (Fe^2+^)) and Cu (cuprous state (Cu^+^) and cupric state (Cu^2+^)), it is stable as a divalent cation (Zn^2+^) and does not directly undergo redox reactions owing to its filled d-shell [93,94]. Zn^2+^ regulates the redox status as an essential component in Cu/Zn SOD and antioxidants via the control of cellular signal transduction, such as gene regulation (e.g., p53, NF-κB, and AP-1) and enzymatic activities [94,95].

Besides the antioxidant properties of Zn, its involvement in various pathways during PD pathogenesis has been overviewed in detail by Aziz et al., 2021 [96]. In brief, after exposure to *P. gingivalis* LPS, an acute phase response is initiated, causing the upregulation of metallothionein expression, which leads to a subsequent deficiency of Zn. This deficiency leads to increased permeability of the gingival epithelium, as well as ineffective NETosis. Both these conditions increase the host’s susceptibility to further infection by periodontal bacteria. In addition, Zn deficiency and LPS favor an increase in M1/M2 macrophage polarization. Because of decreased M2, alveolar bone loss increases and tissue regeneration is impaired, while increased M1 leads to sustained chronic inflammation. LPS also causes a decrease in the transporter ZIP10 expression, leading to a decrease in cytosolic Zn in dendritic and B cells, which weakens the humoral immune response.

There are only a few studies looking into the correlation between Zn levels and periodontal status. The aforementioned cross-sectional study by Freeland et al., 1976 [27], which examined 80 non-fasting individuals, reported that, in contrast with Cu, serum Zn was not correlated with the periodontal index (PI). Similarly, another cross-sectional study by Hamasaki and co-workers, 2017 [90], which examined 3043 participants using linked data from the 2005 National Health and Nutrition Survey, the Comprehensive Survey of Living Conditions, and the Survey of Dental Diseases in Japan, found that according to univariate analyses, there was no significant difference regarding Zn between subjects in the group with CPI 3–4 compared to those with CPI 0–2. In concordance with these results, a more recent case–control study by Yu et al., 2025 [91], which examined Zn concentrations in the serum of 4784 periodontitis cases and 272,252 controls, also found no significant association between Zn and periodontitis, while the Mendelian randomization study by Gao and co-workers [33] also observed no significant causal association between Zn and the risk of periodontitis. On the contrary, Frithiof et al., 1980 [97], examined serum Zn levels of 51 subjects in relation to the severity of PD. Patients with decreased Zn levels showed increased alveolar bone resorption, which led the authors to suggest a possible role of Zn in PD. However, the very small number of participants in this study compromised the extraction of solid conclusions. Finally, the study by Sari et al., 2024 [98], recruited 90 patients, who were equally divided into three groups: periodontitis, gingivitis, and healthy. Serum Zn and total antioxidant status values were significantly higher in the healthy subjects [91 (62–102) μg/dL] compared to those with gingivitis [77 (57–86.5) μg/dL] or periodontitis [69 (54–80.3) μg/dL]. The researchers concluded that Zn deficiency may be linked to an increased severity of periodontitis and elevated oxidative stress levels. Despite the small sample size, one of the advantages of this study is the use of the periodontal inflamed surface area (PISA), which more accurately estimates the extent of periodontal inflammation.

With regard to NHANES studies, the cross-sectional study by Chen and co-workers [24] found that Zn was not significantly associated with chronic periodontitis. However, Xiang et al., 2024 [99], examined the relationship between periodontitis and dietary intake of Zn, based on two 24 h dietary recall surveys in 1914 diabetic patients, and, after adjusting for confounders, demonstrated that subjects who had reached the recommended Zn intake level (males: ≥11 mg/day, females: ≥8 mg/day) had lower odds of periodontitis (OR 0.76, 95% CI: 0.58–0.99). Similarly, Zhou et al., 2024 [100], including 1051 patients from the 2011–2014 NHANES, also concluded that serum zinc levels were associated with the risk of periodontitis in non-diabetic smokers, but not non-smokers.

Although several studies associate the decreased Zn serum levels with more severe PD, there are no investigations assessing the possible beneficial effect of Zn supplementation on periodontal parameters when used as an adjunct to non-surgical PT. The only periodontal intervention study evaluated how PT may affect serum Zn levels in patients with type 2 diabetes [92]. Specifically, non-surgical PT was performed on 120 patients divided into three groups (group 1—patients with periodontitis, group 2—patients with periodontitis and controlled diabetes, and group 3—patients with periodontitis and uncontrolled type 2 diabetes). Intragroup comparisons demonstrated that PT significantly improved serum Zn levels in all groups.

A final remark that should be taken into consideration when interpreting the results of studies evaluating serum levels of Zn is the fact that plasma and serum concentrations account for just 1–2% of the total Zn pool. In addition, infection and stress may shift Zn from plasma to the liver, further reducing its presence in the blood circulation. Thus, serum Zn levels do not necessarily reflect the dietary intake of this mineral [101,102].

### 4.3. Selenium

As an essential trace element, the importance of selenium (Se) in humans is well established, and its deficiency can cause serious health issues, such as cardiomyopathies, compromised immune functions, dysmetabolism of thyroid hormones, and a worse cardiometabolic profile [103,104,105]. Se is an important component of antioxidant enzymes, such as glutathione peroxidase (GPx), thioredoxin reductase (TrxR), and the thyroid-hormone-metabolizing enzymes iodothyronine deiodinases (IDDs) [103]. At least 30 Se-containing proteins (selenoproteins), of which 25 are in humans, have been discovered and exhibit unique physico-chemical properties [106]. Selenocysteine is recognized as the 21st amino acid and forms a predominant residue of selenoproteins and selenoenzymes in biological tissues [107]. The selenoenzymes that are found to have strong antioxidant activity include six groups of the GPx (GPx1, GPx3, GPx4, GPx5, and GPx6). These GPx play a significant role in protecting cells against oxidative damage from ROS and reactive nitrogen species (RNS), which include superoxide, hydrogen peroxide, hydroxyl radicals, nitric oxide, and peroxynitrite [108,109]. The other essential antioxidant selenoenzymes are the TrxR and thioredoxin (Trx), which are used as substrates to hld the Trx/TrxR system in a reduced state for the removal of harmful hydrogen peroxide [110,111]. Major sources of Se are seafood, cereals, and meat products, while lower levels are also found in milk, vegetables, and fruit [112,113].

Very few studies have evaluated the association of PD with Se serum levels. The only cross-sectional study available [114] examined data from a total of 4964 participants from NHANES, who underwent a full-mouth periodontal examination and laboratory tests for five trace minerals, including Se. After adjustment for possible confounders, a significant negative association was observed between blood Se and mean CAL only in males. For people aged 30–44 and without diabetes, the negative association was also statistically significant.

Concerning case–control studies, Thomas et al., 2013 [115], evaluated serum levels of Se of 150 middle-aged subjects of both sexes who were divided into three groups of 50 patients each: group I—patients with type 2 diabetes mellitus and chronic periodontitis, and groups II and III—healthy subjects with and without PD, respectively. Se levels were significantly lower in patients with diabetes and periodontitis compared to healthy controls. Lower, but not statistically significantly, were Se levels in healthy individuals with periodontitis compared to those without periodontitis. Finally, the aforementioned Mendelian randomization study of Gao and co-workers [33] observed no significant causal association between Se and the risk of periodontitis.

Studies looking at the effect of supplementation with Se alone as an adjunct to PT have not been performed, and we must underline further the general scarcity of evidence regarding the role of Se in PD. We should also mention that 24 h recalls are unreliable to estimate the dietary intake of Se, which is strongly affected by the Se content of the soils. On the other hand, the levels of total selenium are unsuitable to estimate the selenium status, since its distribution to serum selenoproteins may vary greatly between individuals [116]. The activity of serum GPX3 seems to be a better surrogate marker of selenium bioavailability. A recent meta-analysis demonstrated increased levels of GPXs in crevicular fluid (Standard Mean Difference: 2.918 ng/μL; CI 95%: 0.372–5.465; *p*-0.025) of periodontitis patients compared to controls, implying the crucial role of this selenoenzyme in combating oxidative stress locally [117].

## 5. Concluding Remarks and Future Directions

Oxidative stress is recognized to play a significant role in PD severity. Therefore, it is reasonable to suggest that a dietary pattern characterized by micronutrients rich in antioxidant properties, such as several vitamins and essential minerals, may positively affect periodontal health. From a nutritional perspective, establishing strong associations between the dietary intake of antioxidants and health outcomes would assist nutritionists in developing tailored dietary regimens for periodontitis patients or individuals at risk for the condition. Additionally, this knowledge could enable pharmaceutical companies to develop targeted nutraceuticals for periodontitis patients, grounded in scientific evidence. However, the evidence from the relevant literature is yet inconclusive.

Studies evaluating the levels of intake of several antioxidant vitamins and essential minerals in relation to periodontal status demonstrate contradictory results. Based on the outcomes of the observational studies, vitamin A demonstrates the most compelling evidence for its ability to reduce the risk of periodontitis, followed by vitamin C. Regarding trace elements, the current level of evidence remains limited, and larger observational studies along with well-designed RCTs are necessary to establish more definitive conclusions. This inconsistency could be attributed to the heterogeneity in the design of the studies and the significant, in some cases, methodological limitations. Since the impact of nutrition on PD is expected to be moderate, studies with a small sample size may lack the necessary statistical power to validate associations. But even the fewer large epidemiological studies present several flaws in their design. Specifically, for practical purposes, some studies evaluate periodontal status based on partial mouth periodontal measurements, which usually underestimate the actual severity of PD. In addition, many studies do not adjust for important risk factors of PD such as smoking, diabetes mellitus, etc., which may have washed out any possible, but weak, effect of nutrition. The accurate assessment of the intake of nutritional antioxidants is also a significant limitation in many studies. Social desirability bias in dietary self-report is very prevalent and may compromise the validity of dietary intake measures. Moreover, when these questionnaires involve only a 24 h recall, the representation of nutritional habits is further compromised. Finally, most of the large observational studies have been conducted in the USA and Asia (primarily Japan and Korea), lacking data from countries with different ethnicities and nutritional backgrounds. Therefore, studies measuring serum levels of these antioxidants may be considered more reliable. However, even in these studies, smoking, diabetes, and inflammation may affect the availability of some antioxidants in blood, while in the case of Zn, serum levels represent only a very small percentage of the total Zn in the body.

Regarding the very few intervention studies that evaluated the effect of nutritional antioxidant supplementation as an adjunct to non-surgical PT, again the results are not consistent. Most of these studies are not randomized, and the dosage and time of use of these supplements vary, rendering comparisons impossible. It should be noted that several intervention studies that use combinations of different nutritional supplements along with PT are available. However, these were not included in this review since the distinction of the actual effect of each nutrient is impossible.

Therefore, if we want, in the future, to improve the evidence and reach solid conclusions, it is necessary to conduct better-designed studies. The assessment methods for both PD and vitamin/mineral intake need to be standardized in order to improve comparability across studies. Prospective longitudinal studies and RCTs are also needed to establish the optimal usage and dosing, causality, as well as long-term effects of vitamin/ mineral supplementation on periodontal health in different patient populations. Last but not least, the integrated application of foodomics with redoxomics, metalloproteomics, and metagenomics will significantly enhance the understanding of the complex relationships between dietary chemical profiles and the pathophysiology of periodontitis, emphasizing the key nutrient regulators of disease severity.

## Figures and Tables

**Table 1:** Dietary reference values according to European Food Safety Authority [11].

VITAMIN/MINERAL	GENDER	AI	AR	PRI	UL
**Vitamin A**	Males	NA	570 μg RE/day	750 μg RE/day	3000 μg RE/day
	Females	NA	490 μg RE/day	650 μg RE/day	3000 μg RE/day
**Vitamin C**	Males	NA	90 mg/day	110 mg/day	ND
	Females	NA	80 mg/day	95 mg/day	ND
**Vitamin E**	Males	13 mg/day	NA	NA	300 mg/day
	Females	11 mg/day	NA	NA	300 mg/day
**Copper**	Males	1.3 mg/day	NA	NA	5 mg/day
	Females	1.3 mg/day	NA	NA	5 mg/day
**Zinc** **(LPI 300 mg/day)**	Males	NA	6.2 mg/day	7.5 mg/day	25 mg/day
	Females	NA	6.2 mg/day	7.5 mg/day	25 mg/day
**Zinc** **(LPI 600 mg/day)**	Males	NA	7.6 mg/day	9.3 mg/day	25 mg/day
	Females	NA	7.6 mg/day	9.3 mg/day	25 mg/day
**Zinc** **(LPI 900 mg/day)**	Males	NA	8.9 mg/day	11 mg/day	25 mg/day
	Females	NA	8.9 mg/day	11 mg/day	25 mg/day
**Zinc (LPI 1200 mg/day)**	Males	NA	10.2 mg/day	12.7 mg/day	25 mg/day
	Females	NA	10.2 mg/day	12.7 mg/day	25 mg/day
**Selenium**	Males	70 μg/day	NA	NA	255 μg/day
	Females	70 μg/day	NA	NA	255 μg/day

**AI:** Adequate Intake, average nutrient level, that is assumed to be adequate for the population’s needs; **AR:** Average Requirement, the intake of a nutrient that meets the daily needs of half the people in a typical healthy population; **PRI:** Population Reference Intake, the intake of a nutrient that is likely to meet the needs of almost all healthy people in a population; **NA:** Not applicable or not assessed; **ND:** Not defined as data were inadequate to derive a value; **UL:** Tolerable upper intake level; the maximum level of total chronic daily intake of a nutrient which is not expected to pose a risk of adverse health effects to humans.

**Table 2:** Summary of cross-sectional studies on the relationship between vitamin A and periodontal disease.

Study	Study Design	Participants	Periodontal Disease Assessment	Vitamin A Assessment	Outcomes
Park et al., 2017 [18]	Cross-sectional	2049 young adults aged 19–39 years (279 periodontitis patients) Country: Korea	CPI greater than or equal to 3 [at least one site had a PPD of >3.5 mm (code > 5.5 mm)]	One 24 h recall	<median vs. >median OR: 1.55 (95% CI: 0.99–2.43) in females
Hosoda et al., 2021 [19]	Cross-sectional	120 Japanese female college students (49 periodontitis patients) Country: Japan	CPI, code 0–4 Periodontitis: CPI code 3–4 No periodontal disease: CPI code 0–2	Self-administered FFQs	Lower intake of beta-carotene in periodontitis patients compared to heathy participants (1515 ± 855 μg/1000 Kcal vs. 2068 ± 1041 μg/1000 Kcal, *p* < 0.05). Multivariate logistic regression analysis: no significant association between vitamin A intake and periodontal disease
Luo et al., 2018 [20]	Cross-sectional	NHANES (2011–2014) 6415 participants No periodontitis: 3465 Moderate disease: 2274 Severe disease: 676 Country: USA	At least two Interproximal sites with PPD ≥ 5 mm not occurring on the same tooth, or at least two interproximal sites that are not on the same tooth and that have CAL ≥4 mm	24 h recall	≤61 vs. ≥786 μg RAE/day OR: 1.78 (95% CI: 1.53–2.39)
Li et al., 2022 [21]	Cross-sectional	NHANES (2009–2014) 6415 participants No periodontitis: 4965 Moderate/severe periodontitis: 3994 Country: USA	≥2 Interproximal sites with CAL ≥4 mm; ≥2 Interproximal sites with PPD ≥5 mm	24 h recall	Sufficient Intake (Males: 900 μg RAE/day, Females: 700 μg RAE/day) vs. Insufficient intake OR: 0.83 (95% CI: 0.69–1.00) The sufficient intake of vitamin A, being able to reduce the risk of periodontitis, was 527 μg RAE/day
Zhou et al., 2023 [22]	Cross-sectional	NHANES (2009–2014) 9081 participants No periodontitis: 5701 Moderate/severe periodontitis: 3380 Country: USA	Moderate periodontitis: ≥2 interproximal sites with PPD ≥5 mm not on the same tooth, or ≥2 interproximal sites with CAL ≥4 mm not on the same tooth; Severe periodontitis: ≥2 interproximal sites with CAL ≥6 mm not on the same tooth and ≥1 interproximal site with PPD ≥5 mm	Two 24 h recalls	2nd vs. 1st tertile of vitamin A intake OR: 0.80 (95% CI: 0.67–0.96) 3rd vs. 1st tertile of vitamin A intake OR: 0.79 (95% CI: 0.65–0.96) The association was still significant in populations who were less than 60 years old, non-Hispanic black, Poverty Index (PI) ≤ 1.3, 1.3 < PI ≤ 3.5, non-smokers, obese, and not exhibiting diabetes mellitus or hypertension
Liang et al., 2024 [23]	Cross-sectional	NHANES (2009–2014) 9820 participants No periodontitis: 6288 Periodontitis: 3532 Country: USA	Mild periodontitis: ≥2 interproximal sites with CAL ≥ 3 mm, and ≥ 2 interproximal sites with PPD ≥ 4 mm (not on the same tooth) or one site with PPD ≥ 5 mm; Moderate periodontitis: 2 interproximal sites with CAL ≥ 4 mm, not on the same tooth, or the presence of at least 2 interproximal sites with PPD ≥5 mm, not on the same tooth; Severe periodontitis: 2 interproximal sites with CAL ≥ 6 mm (not on the same tooth), or ≥1 interproximal sites with PPD ≥ 5 mm (not on the same tooth). Mild, moderate, and severe periodontitis were classified as having periodontitis.	24 h recall	≥528 μg RAE/day vs. <528 μg RAE/day OR: 0.88 (95% CI: 0.76–1.02), *p* = 0.094
Chen et al., 2025 [24]	Cross-sectional	NHANES (2009–2014) 11,704 participants No periodontitis: 6288 Periodontitis: 3532 Country: USA	Mild periodontitis: ≥2 interproximal sites with CAL ≥ 3 mm and ≥2 interproximal sites with PPD ≥ 4 mm, on different teeth, or one site with PPD ≥ 5 mm; Moderate periodontitis: ≥2 interproximal sites with CAL ≥ 4 mm, on different teeth, or ≥2 interproximal sites with PPD ≥ 5 mm, on different teeth; Severe periodontitis: ≥2 interproximal sites with CAL ≥ 6 mm, on different teeth, and ≥1 interproximal site with PPD ≥ 5 mm; No periodontitis: does not meet any of the criteria for periodontitis	Two 24 h recalls	Vitamin A intake was not causally associated with chronic periodontitis
Chapple et al., 2007 [25]	Cross-sectional	NHANES III survey (1988–1994) 11,480 participants Mild periodontitis: 1567 Severe periodontitis: 609 Country: USA	At least one site with both CAL ≥4 mm and PPD of ≥4 mm	Serum α- and β-carotene	Highest vs. lowest quintile of serum α-carotene (mild periodontitis) OR: 0.60 (95% CI: 0.46–0.77) Highest vs. lowest quintile of serum α-carotene (severe periodontitis) OR: 0.85 (95% CI: 0.67–1.07) Highest vs. lowest quintile of serum β-carotene (mild periodontitis) OR: 0.80 (95% CI: 0.65–0.98) Highest vs. lowest quintile of serum β-carotene (severe periodontitis) OR: 0.65 (95% CI: 0.46–0.93)
Linden et al., 2009 [26]	Cross-sectional	1285 old men (60–70 years) County: Northern Ireland	Severe periodontitis >15% of all sites measured had a CAL (6 mm), and there was at least one site with deep pocketing (6 mm)	Serum α- and β-carotene, β-cryptoxanthin and zeaxanthin	Inverse association between α- and β-carotene, β-cryptoxanthin and low-threshold periodontitis. Lowest vs. Highest quintile of β-cryptoxanthin: aOR 4.02 for high-threshold periodontitis

**CAL:** Clinical Attachment Loss; **CPI:** Community Periodontal Index; **NHANES**: National Health and Nutrition Examination Survey; **OR:** odds ratio; **PI:** Poverty Index; **PPD:** Probing Pocket Depth; **RAE:** Retinol Activity Equivalents.

**Table 3:** Cross-sectional studies on the relationship between vitamin C and periodontal disease.

Study	Study Design	Participants	Periodontal Disease Assessment	Vitamin C Assessment	Outcomes
Park et al., 2017 [18]	Cross-sectional	2049 young adults aged 19–39 years (279 periodontitis patients) Country: Korea	CPI greater than or equal to 3 [at least one site had a probing pocket depth of >3.5 mm (code > 5.5 mm)]	One 24 h recall	<median vs. >median OR: 1.66 (95% CI: 1.04–2.64) in females and OR: 1.49 (95% CI: 1.04–2.14) in current non-smokers
Luo et al., 2018 [20]	Cross-sectional	NHANES (2011–2014) 6415 participants No periodontitis: 3465 Moderate disease: 2274 Severe disease: 676 Country: USA	At least two Interproximal sites with PPD ≥ 5 mm not occurring on the same tooth, or at least two interproximal sites that are not on the same tooth and that have CAL ≥4 mm	24 h recall	Vitamin C intake ≤20.65 vs. ≥112.91 mg/day; aOR = 1.401 (95% CI = 1.12–1.74)
Li et al., 2022 [21]	Cross-sectional	NHANES (2009–2014) 6415 participants No periodontitis: 4965 Moderate/severe periodontitis: 3994 Country: USA	≥2 Interproximal sites with CAL ≥4 mm; ≥2 Interproximal sites with a PPD of ≥5 mm	24 h recall	Sufficient (Males: >90 mg/day, Females: >70 mg/day) vs. Insufficient intake OR: 1.13 (95% CI: 1.03–1.23) Excessive vitamin C intake was linearly and positively correlated with an increased risk of periodontitis
Liang et al., 2024 [23]	Cross-sectional	NHANES (2009–2014) 9820 participants No periodontitis: 6288 Periodontitis: 3532 Country: USA	Mild periodontitis: ≥2 interproximal sites with CAL ≥ 3 mm, and ≥2 interproximal sites with PPD ≥ 4 mm (not on the same tooth) or one site with PPD ≥ 5 mm; Moderate periodontitis: 2 interproximal sites with CAL ≥ 4 mm, not on the same tooth, or the presence of at least 2 interproximal sites with PPD ≥5 mm, not on the same tooth; Severe periodontitis: 2 interproximal sites with CAL ≥ 6 mm (not on the same tooth), or ≥1 interproximal sites with PPD ≥ 5 mm (not on the same tooth). Mild, moderate, and severe periodontitis were classified as having periodontitis.	24 h recall	≥89.4 mg/day vs. <89.4 mg/day OR: 0.93 (95% CI: 0.82–1.04), *p* = 0.218
Chen et al., 2025 [24]	Cross-sectional	NHANES (2009–2014) 11,704 participants No periodontitis: 6288 Periodontitis: 3532 Country: USA	Mild periodontitis: ≥2 interproximal sites with CAL ≥ 3 mm and ≥2 interproximal sites with PPD ≥ 4 mm, on different teeth, or one site with PPD ≥ 5 mm; Moderate periodontitis: ≥2 interproximal sites with CAL ≥ 4 mm, on different teeth, or ≥2 interproximal sites with PPD ≥ 5 mm, on different teeth; Severe periodontitis: ≥2 interproximal sites with CAL ≥ 6 mm, on different teeth, and ≥1 interproximal site with PPD ≥ 5 mm; No periodontitis: does not meet any of the criteria for periodontitis	Two 24 h recalls	Vitamin C intake was not causally associated with chronic periodontitis
Chapple et al., 2007 [25]	Cross-sectional	NHANES III survey (1988–1994) 11,480 participants Mild periodontitis: 1567 Severe periodontitis: 609 Country: USA	At least one site with both CAL ≥4 mm and PPD of ≥4 mm	Serum vitamin C	Serum vitamin C concentration: highest (>70.41 mmol/L) vs. lowest (<8.52 mmol/L); aOR = 0.53 (95% CI = 0.42–0.68)
Lee et al., 2017 [49]	Cross-sectional	KNHANES IV 10,930 adults (≥19 years) Country: Korea	CPI score; Periodontitis: CPI = 3 or 4	24 h dietary record	Lowest intake (<47.3 mg/day) vs. highest intake (≥132.2 mg/day) aOR: 1.28 (95% CI = 1.10–1.50)
Nishida et al., 2000 [51]	Cross-sectional	NHANES III survey (1988–1994) 12,419 adults (>20 years of age) Country: USA	Clinical attachment level ≥1.5: periodontal disease	24 h dietary record	Vitamin C intake (<0–29 mg/day) vs. (>180 mg/day); aOR = 1.30
Amarasena et al., 2005 [54]	Cross-sectional	413 elderly citizens (70 years) of Niigata Country: Japan	CAL (six sites of all teeth present including third molars)	Serum vitamin C levels	Serum vitamin C concentration was inversely related to CAL (r = –0.23, *p* < 0.00005) at bivariate level.

**CI:** Confidence Interval; **CAL:** Clinical Attachment Loss; **CPI:** Community Periodontal Index; **KNHANES**: Korea National Health and Nutrition Examination Survey; **NHANES**: National Health and Nutrition Examination Survey; **OR:** Odds Ratio; **PPD:** Probing Pocket Depth.

**Table 4:** Cross-sectional studies on the relationship between vitamin E and periodontal disease.

Study	Study Design	Participants	Periodontal Disease Assessment
Luo et al., 2018 [20]	Cross-sectional	NHANES (2011–2014) 6415 participants No periodontitis: 3465 Moderate disease: 2274 Severe disease: 676 Country: USA	At least two Interproximal sites with PPD ≥ 5 mm not occurring on the same tooth, or at least two interproximal sites that are not on the same tooth and that have CAL ≥4 mm
Li et al., 2022 [21]	Cross-sectional	NHANES (2009–2014) 6415 participants No periodontitis: 4965 Moderate/severe periodontitis: 3994 Country: USA	≥2 Interproximal sites with CAL ≥4 mm; ≥2 Interproximal sites with PPD ≥5 mm
Chapple et al., 2007 [25]	Cross-sectional	NHANES III survey (1988–1994) 11,480 participants Mild periodontitis: 1567 Severe periodontitis: 609 Country: USA	At least one site with both CAL ≥4 mm and PPD of ≥4 mm
Zong et al., 2015 [82]	Cross-sectional	NHANES (1999–2001) 4708 participants Country: USA	Mean CAL, mean PPD, and periodontitis were calculated using data collected at mesiobuccal sites for consistency between survey circles, and interproximal sites are more reflective of periodontitis than midbuccal sites. Total Periodontitis (TPD) was defined as the sum of mild, moderate, and severe periodontitis according to CDC

**aT:** α-tocopherol; **CAL:** Clinical Attachment Loss; **CI:** Confidence Interval; **OR:** Odds Ratio; **PPD:** Probing Pocket Depth; **TC:** Total Cholesterol; **TPD:** Total Periodontitis.

## Abbreviations

The following abbreviations are used in this manuscript:

PD	Periodontal disease
PT	Periodontal treatment
PPD	Probing pocket depth
CAL	Clinical attachment loss
PI	Periodontal index
CPI	Community Periodontal Index
CPITN	Community Periodontal Index of Treatment Needs
PISA	Periodontal inflamed surface area
FFQ	Food frequency questionnaire
AscH_2_	Ascorbic acid
AscH	Ascorbate monoanion
RCT	Randomized clinical trial
TPD	Total periodontitis
ROS	Reactive oxygen species
MDA	Malondialdehyde
NO	Nitric oxide
8-OH-dG	8-hydroxy-deoxyguanosine
DHA	Dehydroascorbic acid
OR	Odds ratio
CI	Confidence Interval
aT	a-tocopherol
GWAS	Genome-wide association study
Cu	Copper
Mn	Manganese
Zn	Zinc
TC	Total cholesterol
h	hours
HGFs	human gingival fibroblasts
SRP	scaling and root planing
Se	Selenium
GPx	Glutathione peroxidase
TrxR	Thioredoxin reductase
IDD	Iodothyronine deiodinases
Trx	Thioredoxin
MR	Mendelian Randomization
BMI	Body Mass Index
IVW	Inverse Variance Weighted
